# Cooperation under Indirect Reciprocity and Imitative Trust

**DOI:** 10.1371/journal.pone.0013475

**Published:** 2010-10-27

**Authors:** Serguei Saavedra, David Smith, Felix Reed-Tsochas

**Affiliations:** 1 Northwestern Institute on Complex Systems, Northwestern University, Evanston, Illinois, United States of America; 2 Kellogg School of Management, Northwestern University, Evanston, Illinois, United States of America; 3 CABDyN Complexity Centre, Oxford University, Oxford, United Kingdom; 4 Oxford Centre for Integrated Systems Biology, Oxford University, Oxford, United Kingdom; 5 Centre for Mathematical Biology, Oxford University, Oxford, United Kingdom; 6 Institute for Science, Innovation, and Society, Saïd Business School, Oxford University, Oxford, United Kingdom; University of Zurich, Switzerland

## Abstract

Indirect reciprocity, a key concept in behavioral experiments and evolutionary game theory, provides a mechanism that allows reciprocal altruism to emerge in a population of self-regarding individuals even when repeated interactions between pairs of actors are unlikely. Recent empirical evidence show that humans typically follow complex assessment strategies involving both reciprocity and social imitation when making cooperative decisions. However, currently, we have no systematic understanding of how imitation, a mechanism that may also generate negative effects via a process of cumulative advantage, affects cooperation when repeated interactions are unlikely or information about a recipient's reputation is unavailable. Here we extend existing evolutionary models, which use an image score for reputation to track how individuals cooperate by contributing resources, by introducing a new imitative-trust score, which tracks whether actors have been the recipients of cooperation in the past. We show that imitative trust can co-exist with indirect reciprocity mechanisms up to a threshold and then cooperation reverses -revealing the elusive nature of cooperation. Moreover, we find that when information about a recipient's reputation is limited, trusting the action of third parties towards her (i.e. imitating) does favor a higher collective cooperation compared to random-trusting and share-alike mechanisms. We believe these results shed new light on the factors favoring social imitation as an adaptive mechanism in populations of cooperating social actors.

## Introduction

The evolution of cooperative behavior in biological and human populations has been shown to rely critically on different forms of reciprocity [1]–[6]. In human society, cultural transmission mechanisms such as language allow for a subtle cooperative structure based on the principle of indirect reciprocity. In the absence of previous direct interactions which can be used to judge an individual, it is possible to observe and record the interactions of that individual with third parties [7], [8], and assign a reputation to the individual guided by the principle: if I scratch your back, someone else will scratch mine [9]. Simulation models in which a reputation score associated with each actor records previous decisions about whether to cooperate or not, have revealed that indirect reciprocity among actors in a population will emerge particularly if all individuals have access to the reputation scores of other individuals [10]–[12].

However, when information about the past record of other individuals is unavailable or unreliable, laboratory experiments [13]–[20] and simulation models [14], [21]–[23] have shown that actors might rely instead on imitation mechanisms or recognition heuristics to share resources with other actors they interact with according to their counterpart's trustworthiness. In fact, recent work has shown that cooperative behavior can spread as an imitation and trust mechanism across a population of self-regarding individuals [24]. The trustworthiness can be assigned to actors on the basis of how many third parties signal that they endorse a given actor, and as such is used as a proxy for the attributes of an individual when there is no detailed record of how those actors have acted towards others in the past [17], [18], [25], [26]. This is to say, an actor C will extend trust to A (i.e. cooperate with A), because B previously extended trust to A, and in the absence of further information the trustworthiness of A can be used as part of a frugal heuristic or referral mechanisms by C [20], [24], [27], [28].

Although reliance on imitation strategies can provide a heuristic that allows the identification of potentially trustworthy partners in interactions, there can be a negative impact on overall welfare since the resulting distribution of resources can reflect the principle of cumulative advantage [25], [29]–[31]. Following this principle implies that the distribution of resources across actors in a population becomes increasingly skewed over time, with the rich getting richer and the poor getting poorer. Similarly, imitation strategies have proved extremely successful when applied to competitive strategies [32], [33]. Here we explore how actors use different assessment attributes based on imitation and indirect reciprocity mechanisms to decide whom they cooperate with, and who gains resources when repeated interactions are unlikely. We answer the questions of whether imitation and indirect reciprocity mechanisms can co-exist and generate collective cooperation, and whether imitation provides a reliable alternative to indirect reciprocity when information about an actor's reputation is frequently unavailable. In general, why is imitation a recurrent mechanism in human behavior given its potential negative effects on the distribution of resources in a population?

## Results

### The model

In our imitation-reciprocity (IR) model, we consider individuals faced with a social dilemma [1], [9], [34], [35], who follow cooperative or altruistic strategies involving both reciprocity and imitation mechanisms [24], [27], [28]. Here, the donor has the opportunity to help a randomly chosen recipient at cost 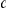, while the recipient gets a benefit 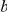. Otherwise, the donor and recipient remain with their current payoff [10]. Hence, the donor faces a dilemma about whether to cooperate or not. However, we assume that non-cooperative actions harm the reputation and trust of the donor and recipient respectively. For reputation, we follow the image-scoring mechanism proposed in [10], where the image 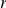 of a donor is continually assessed according to their previous cooperative or non-cooperative actions towards other possible recipients in the population. Similarly, for imitative trust, a recipient's image 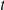 is continually assessed according to the cooperative or non-cooperative actions received from possible donors. Hence, the trust score of a recipient only records information about the action of third parties towards her. Donors have their own assessment strategies 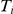 and 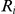 for trust and reputation images respectively. A positive image of a recipient 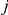 always will make it more likely that a donor 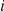 will help than a negative image given by 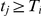 or 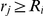. This corresponds to the behavior of actors who have access to the reputation of potential recipients, and social actors using imitative strategies, who only have access to or are influenced by the trustworthiness of such recipients. The access to information is given by a threshold parameter 
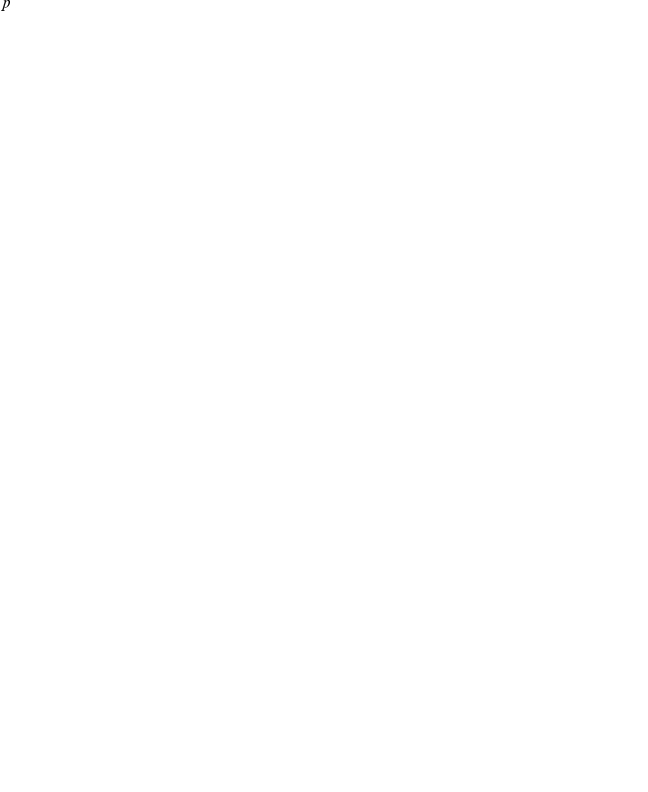
, which determines whether donors evaluate the reputation, with probability 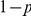, or the trustworthiness of recipients, with a probability 
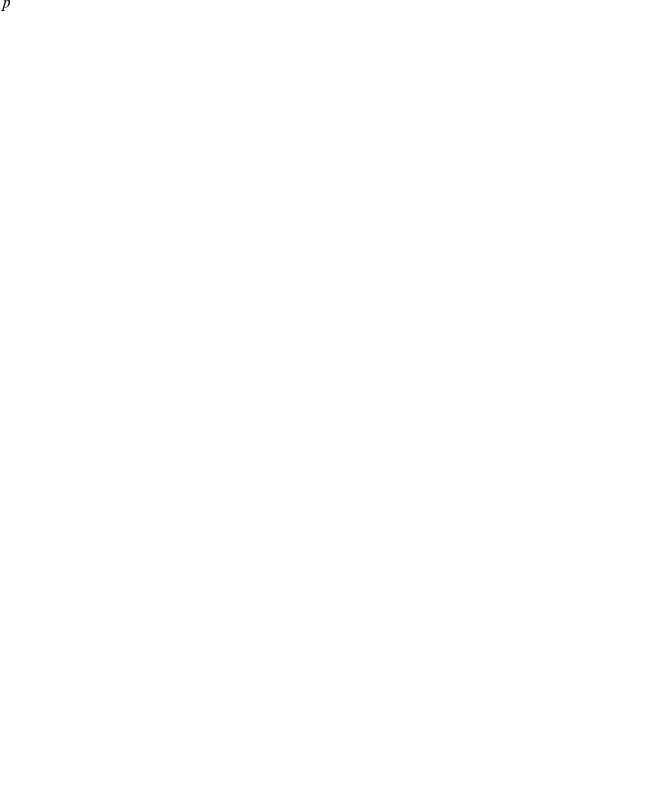
. In our simulations, we consider 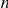 actors, which are replaced at the end of each generation 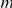 and transmit their strategies to the new population in proportion to their accumulated payoffs (Methods). In each generation, 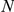 randomly pair-wise interactions are chosen, where actors can play either the role of donors or recipients, i.e. 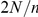 interactions per actor (see Methods for a detailed description of the IR model).

### Imitation and indirect reciprocity

**Figure 1:**
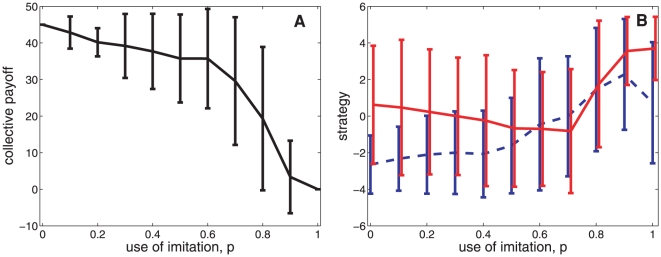
Imitative trust and indirect reciprocity. Panel **A** and **B** show, respectively, the average payoff per actor and the average strategies 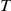 (solid red line) and 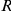 (dashed blue line) observed in the population across different levels of imitation 
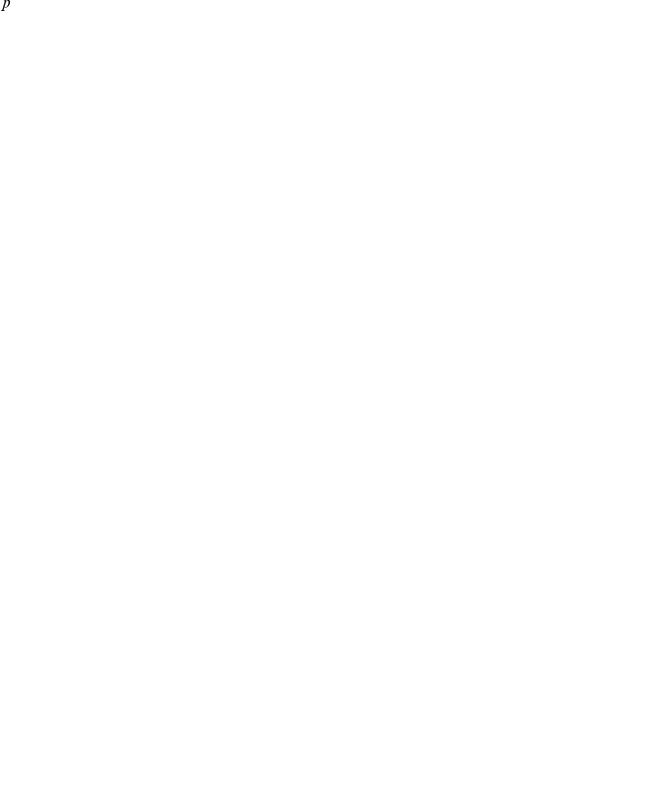
 (x-axis). Bars correspond to 2 standard deviations. Values are calculated over 1000 simulations considering the generation when the population has reached a fixated common strategy 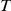, 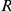.

First, we analyze the effects of using imitative trust as an alternative mechanism to indirect reciprocity. We find that the collective payoff generated by indirect reciprocity is surprisingly robust to high levels of imitation. As illustrated in Figure 1A, we find that for most of the simulated levels of imitation 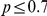, the average payoff per actor, calculated across the generation once the population has fixated into a common strategy, is higher than half of the maximum possible (i.e. payoff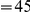). However, the average payoff considerably decreases as imitation becomes the only strategy followed by actors (i.e. payoff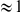), revealing the elusive nature of cooperation. Similarly, analyzing the fixated strategies reached by the population in the last generation, we find that on average both imitative 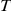 (solid red line) and indirect reciprocity 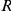 (blue dashed line) strategies become non-cooperative (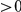) at a high level of imitation 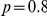 (see Fig. 1B). This suggests that only when imitation is used less than 80% as an assessment strategy, cooperative behavior dominates and the population achieve higher payoffs.

**Figure 2:**
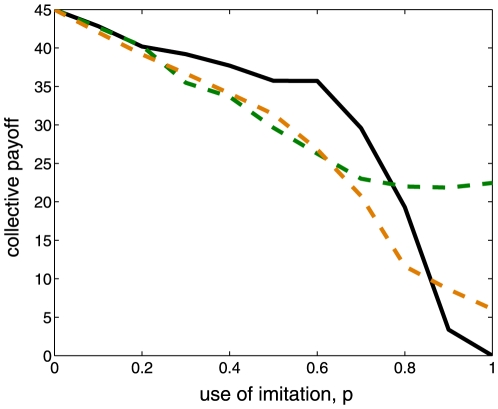
Alternative cooperative mechanisms to imitation. The figure compares the average payoff per actor (see Fig. 1A) generated by imitative trust (black line) against the average payoff obtained by replacing imitative trust with random-trusting (green dashed line) and share-alike (red dashed line) mechanisms. Values are calculated over 1000 simulations considering the generation when the population has reached a fixated common strategy 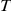, 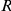.

Additionally, we explore to what extent trusting the actions of others provides better cooperative outcomes than plausible alternative strategies. Our first alternative strategy or null hypothesis is a random-trusting process, where we assume that donors apply a simple probabilistic rule and cooperate on average 50% of the time. This is to say, when information about a recipient's reputation is unavailable 
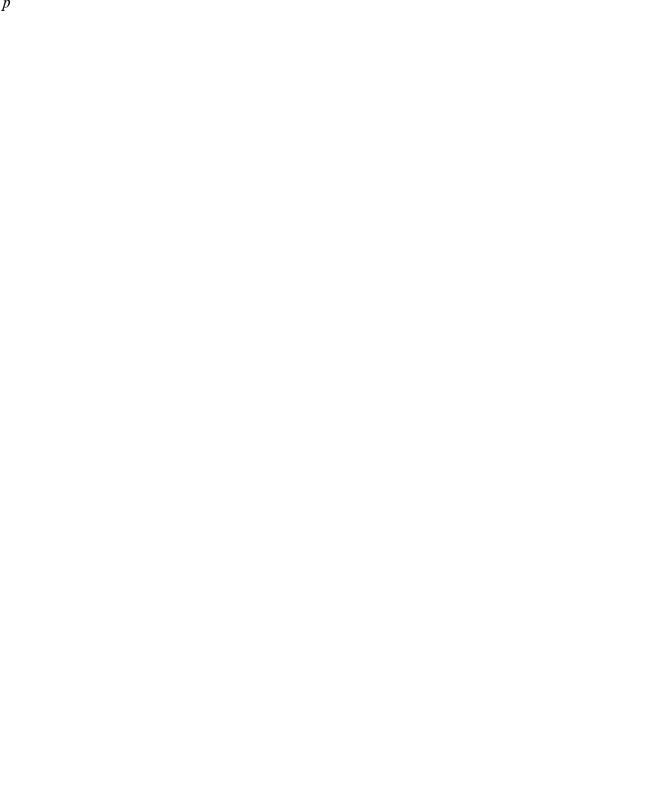
 percent of the time, donors apply a simple random process and cooperate on average one out of two opportunities. For the second null hypothesis, we assume that donors follow a share-alike behavior, where they try to distribute benefits equally among all members in the population [36], [37]. Here, donors cooperate if the trustworthiness (i.e. previous granted cooperation) of the recipient is low and defect if the trustworthiness is high (see Methods for details). Figure 2 shows that under intermediate levels of limited information 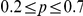, random-trusting (green dashed line) processes and share-alike mechanisms (orange dashed line) display on average lower payoffs than imitative trust (black line). This reveals that trusting the action of others could be a useful alternative mechanism to indirect reciprocity when donors do not have frequent access to the reputation of potential recipients.

### Vulnerability of cooperative strategies

To examine the vulnerability of different strategies on distributing equal number of resources to all members in the population, we investigate the effect of noise in the allocation of resources. We measure the distribution of payoffs in the population generated by changing the parameter 
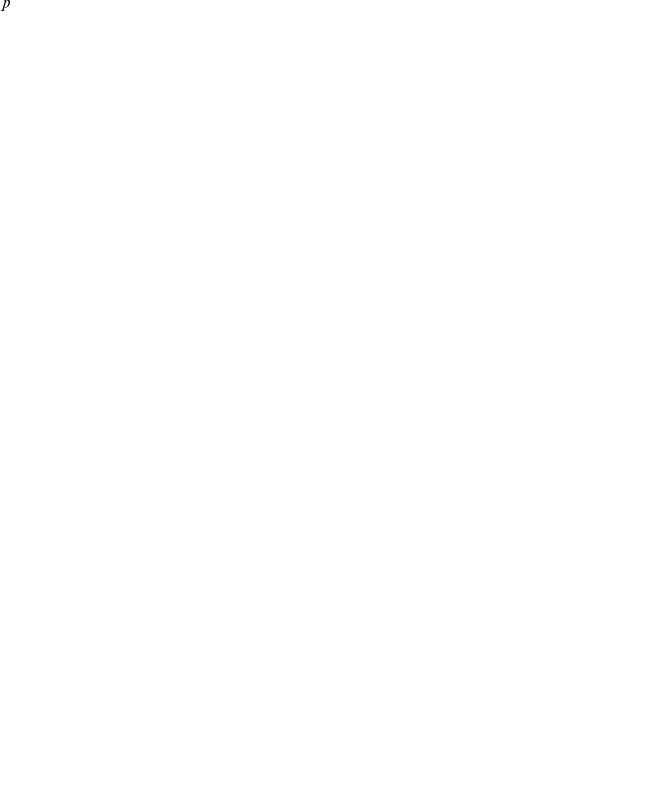
 and introducing small errors in the decision-making process of donors [11]. This noise in the allocation of resources takes into account important effects such as memory constraints, bias in judgments or implementation errors [38]–[42]. We implement this by allowing donors to randomly change their decision with a small, fixed probability 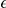 [11]. Here we consider that one out of ten times a donor can make an implementation or decision error (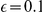). Smaller values of 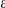 generate similar results. Note that without this noise we would expect all actors with the same amount of resources.

**Figure 3:**
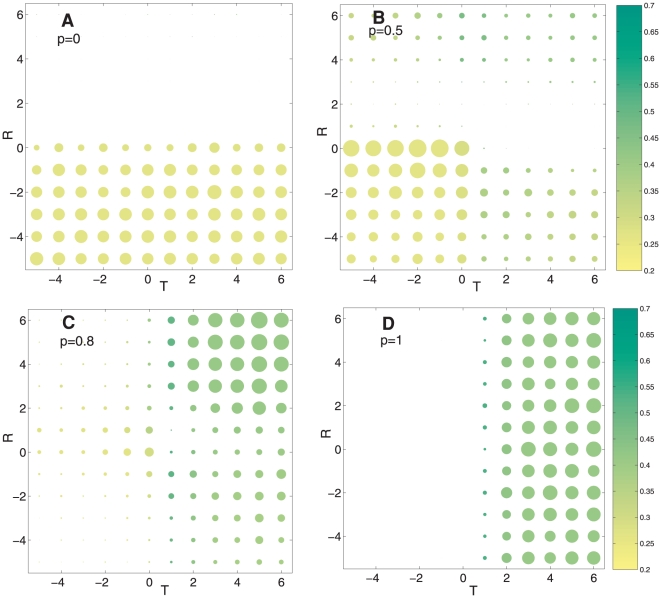
Vulnerability of cooperative strategies. We analyze the vulnerability of imitation and indirect reciprocity strategies on distributing similar resources when actors are subject to implementation errors. We introduce a probability 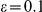 that donors mistakenly act in the opposite way as it was expected from their strategy. Note that without errors we would expect all actors with the same amount of resources. Panels **A–D** show the correlation between Gini coefficients (shades) and the frequency of fixated strategies (circles) for 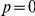, 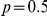, 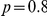 and 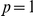 respectively. Gini coefficients and frequencies are reported as the average over 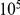 simulations considering the generation when the population has reached a fixated common strategy 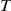, 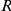. The frequency of occurrence for each strategy is proportional to the area of the circles.

To measure the distribution of payoffs in the population, we use the Gini coefficient [43]. The Gini coefficient represents the average difference in wealth share for two actors in the population normalized to fall between 0 (perfect equality) and 1 (maximum inequality). The Gini coefficient is defined as 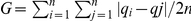, where 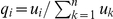, and 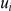 is the payoff of actor 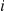, and 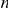 is the total number of actors in the population. Since payoffs can be negative, we take the minimum value as the baseline payoff equal to 1 and adjust all other payoffs accordingly. Figure 3A shows that under pure indirect reciprocity (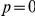), the population always favors cooperative strategies 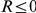 with low Gini coefficients. Interestingly, Figure 3B shows that even when imitation and indirect reciprocity mechanisms are used equally 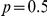, the population has a high likelihood (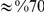) of converging into cooperative strategies with low Gini coefficients (bottom left corner). By contrast, Figures 3C–D show that cooperators disappear and high Gini coefficients emerge at the point when imitation dominates the assessment mechanism in the population. Note that the highest Gini coefficients are reached when the population follows a trust-based cooperative strategy 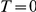 combined with a reputation-based unconditional defector strategy 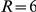. This shows that populations that only cooperate using imitation mechanisms are highly prone to inequality effects [25], [29]–[31].

### Emergence of cooperation

Finally, we investigate whether imitative trust and indirect reciprocity can co-exist and allow the emergence of reciprocal altruism. We explore how cooperation would evolve through mutations in a population of unconditional defectors. For each actor, we introduce a third dimension 
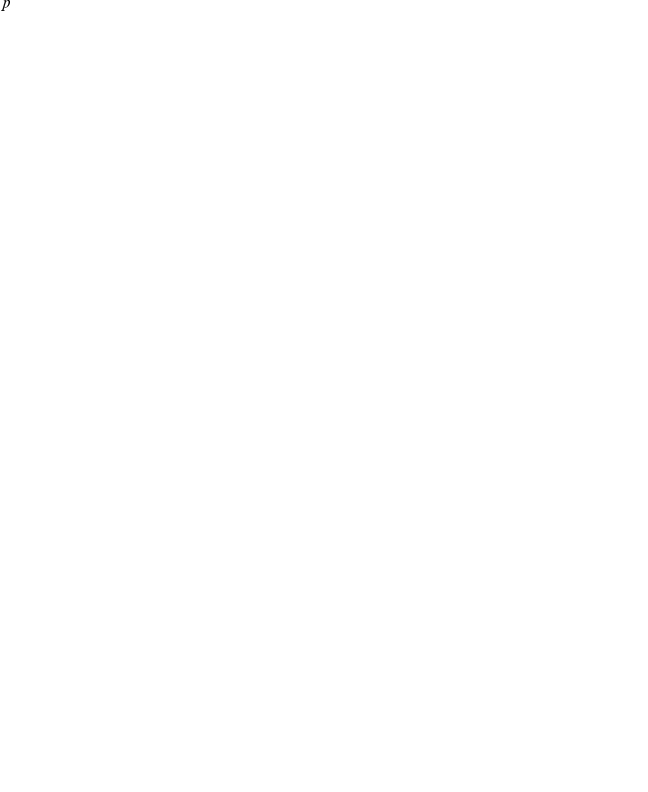
. For simplicity we assume it can take three different values 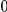, 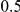 and 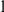, which correspond to the proportion of imitative trust used by actors, i.e. this dimension replaces the probability of using imitative trust caused by limited information in our original model (see Methods). We initialize the population with all actors having 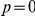, 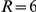 and a random strategy 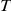, i.e. at the beginning actors only use indirect reciprocity strategies defined by unconditional defectors. To investigate the evolution of imitative trust and cooperation, we include mutations in the creation of new generations. We assume a small probability 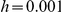 [10] that a new actor adopts a randomly chosen strategy (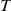, 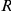 and 
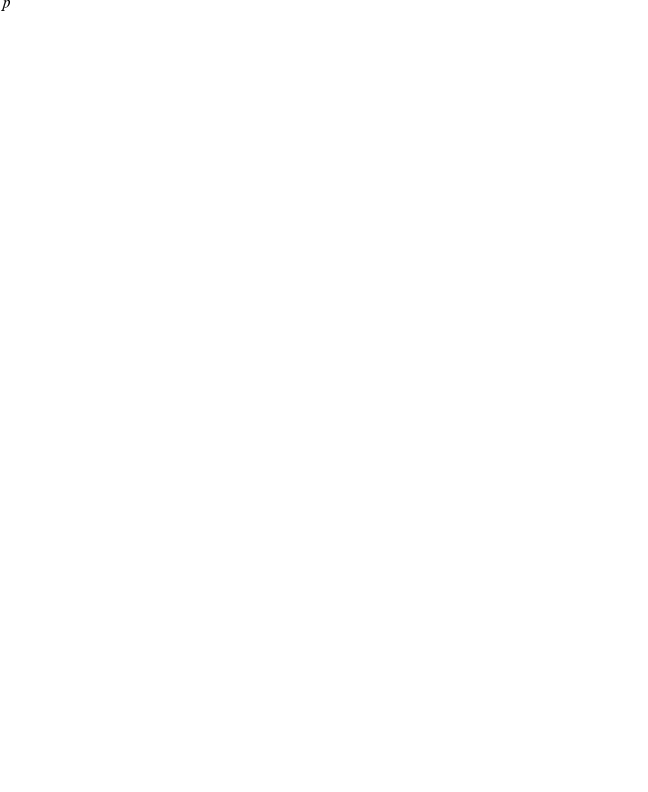
) than the one inherited by her parent.

**Figure 4:**
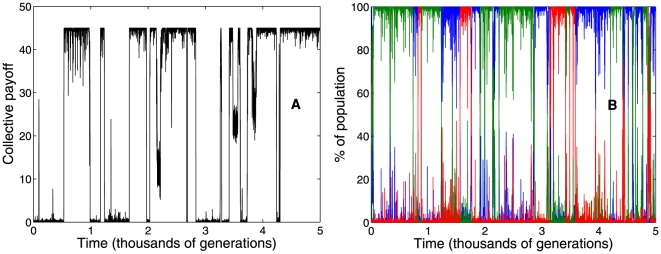
Emergence of cooperation. To investigate the emergence of cooperation and the co-existence of imitation and indirect reciprocity, we consider that new actors will adopt a randomly chosen strategy with probability 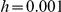 (see text). Additionally, to differentiate between actors using only indirect reciprocity, imitation or a mix of the two, we introduce a third dimension 
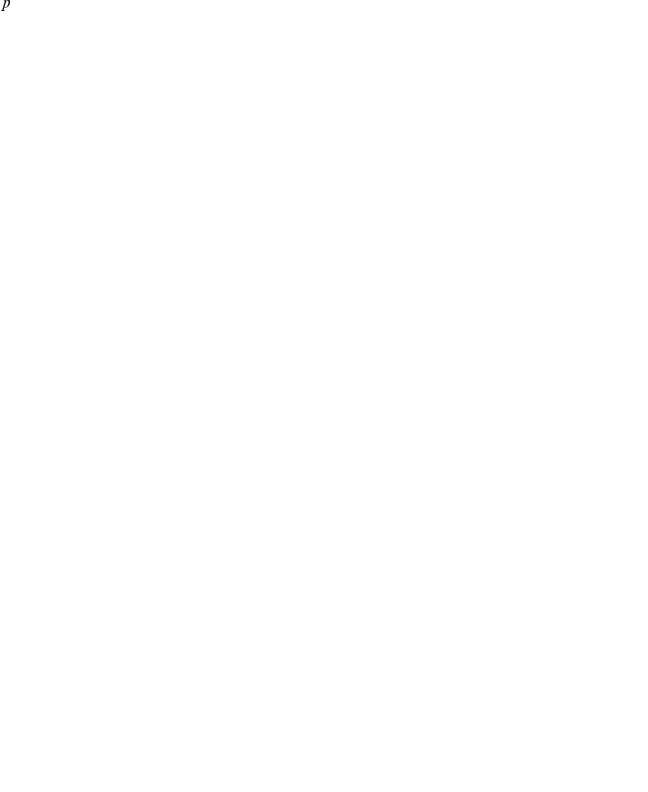
 that takes values of 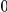 (blue line), 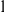 (red line), or 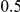 (green line) respectively. We initialize the population with 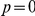 and 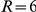, i.e. unconditional defectors. Panel **A** shows the average payoff per actors per generation for a single simulation. Note that the population continuously fluctuate between maximum cooperation and defection. Panel **B** shows that the strategies 
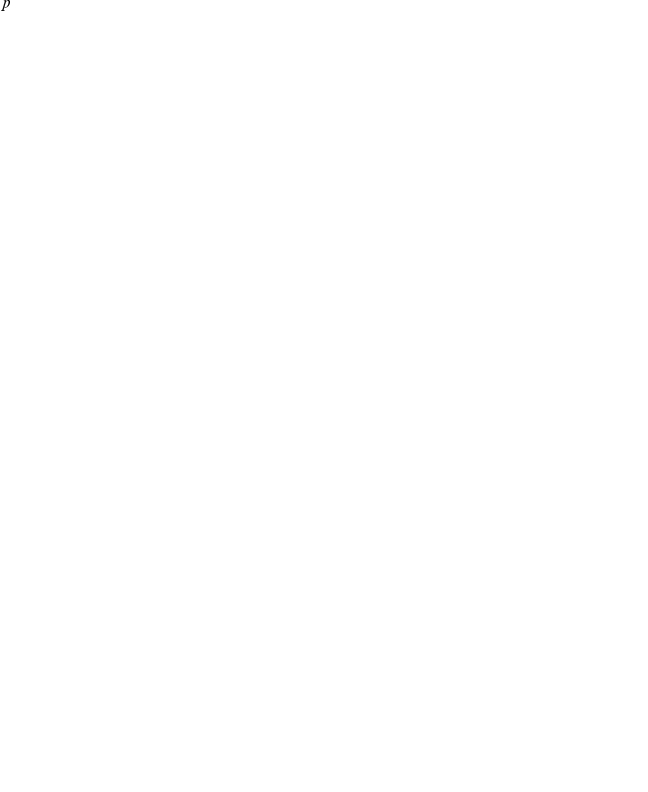
 also fluctuate across generations. This reveals that although there is no stable strategy, actors can adopt cooperative imitative and indirect reciprocity strategies.

First, our simulations show endless cycles of collective cooperation and defection. Figure 4A shows that the average payoff per actor per generation continuously fluctuates between 45, the maximum value, and 0, the minimum value. Second, we observe that imitative trust can, in fact, co-exist with indirect reciprocity. Figure 4B shows the percentage of actors with either 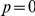 (blue), 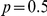 (green) and 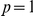 (red) across thousands of generations for a single simulation. The population continuously fluctuates between all the different strategies. Similar results hold if we only use 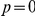 and 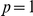. Note that the population never settles in a stable strategy. These results suggest that cooperation emerges only if indirect reciprocity is present; however, once this requirement is fulfilled, imitation can provide a plausible alternative strategy.

## Discussion

It has been argued that cultural transmission mechanisms make it possible to assign a measure of reputation or social status to specific individuals in a population, so that cooperation can emerge in human societies as a consequence of indirect reciprocity [9], [10]. However, the effects of reputation and social status do not necessarily coincide, and therefore are likely to warrant separate treatment. The evaluation of reputation takes into account the record of past actions of an individual, while social status reflects social preferences and mechanisms such as copying the helping behaviour of others [44]. Social experiments have shown that the actions and opinions of others used as a proxy for quality or reputation can affect someone's popularity or commitment to cooperate [18], [24]. If we assume that actors are heterogeneous in this regard, then it is useful to model populations so that individuals vary in how they attend to these two types of information, and hence to allow for different combinations of reputation and imitative trust mechanisms. Although there is no difference in principle with regard to the cognitive demands imposed by each mechanism, there may also be asymmetries between the availability or quality of information associated with giving help and receiving help in a given social setting. Methodologically, the addition of imitative trust to the original indirect reciprocity model restores balance to how information on donors and recipients is treated. Each pairwise interaction between a donor and recipient encodes information about both parties, which the combination of image and imitative-trust scores fully captures.

Here we have analyzed for the first time the effects that two assessment mechanisms –imitation and indirect reciprocity, which determine the structure of who cooperates with whom and who gains resources, might generate when access to the reputation of potential recipients is frequently unavailable or actors are influenced by the cooperative action of others. We have found that both the cooperative behavior and the fair allocation of resources decrease as the use of imitation mechanisms increases. However, we have also found that as long as actors use imitation and indirect reciprocity mechanisms equally, cooperative strategies dominate and resource inequalities are small. Surprisingly, we have observed that trusting the action of others generates higher payoffs than simple random-trusting processes and share-alike mechanisms. This suggests that imitation might be in fact an adaptive mechanism in populations of cooperating social actors under limited information.

## Materials and Methods

### IR Model

We study reciprocal altruism under imitative trust and indirect reciprocity. Specifically, we consider 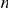 actors over a fixed lifetime, which are replaced at the end of each generation 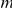. In each generation, 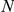 randomly pair-wise interactions are chosen, where actors can play either the role of donors or recipients (i.e. 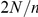 interactions per actor). If a donor 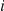 cooperates with a recipient 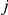, the donor pays a cost 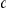 and the recipient gets a benefit 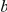. Otherwise, if the donor does not cooperate, both payoffs remain exactly the same. A donor 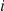 decides whether to cooperate or not based on the recipient's image and her own assessment strategy. The image of a recipient 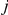 is assessed either by her trust score 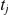 or reputation score 
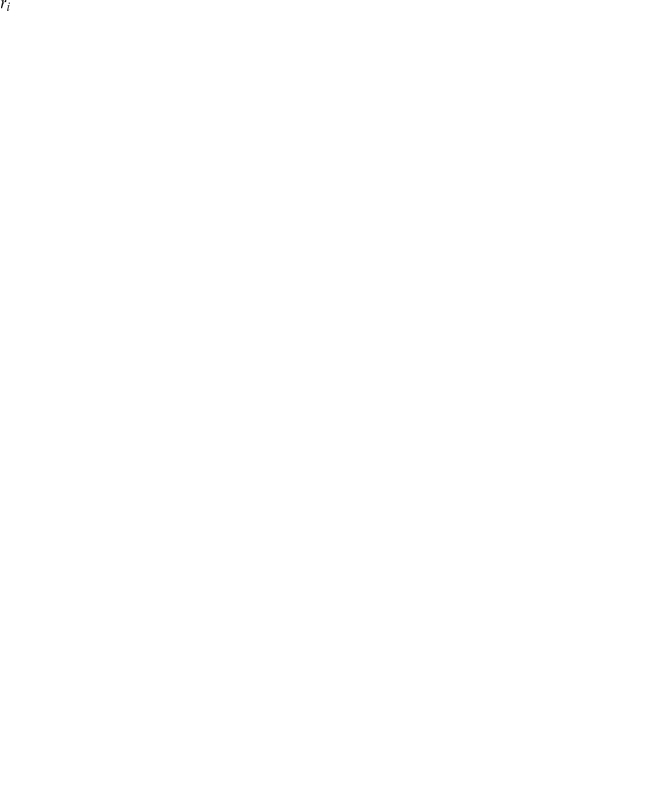
, where both can take integer values in 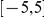 following the standard convention of reference [10]. A tunable parameter 
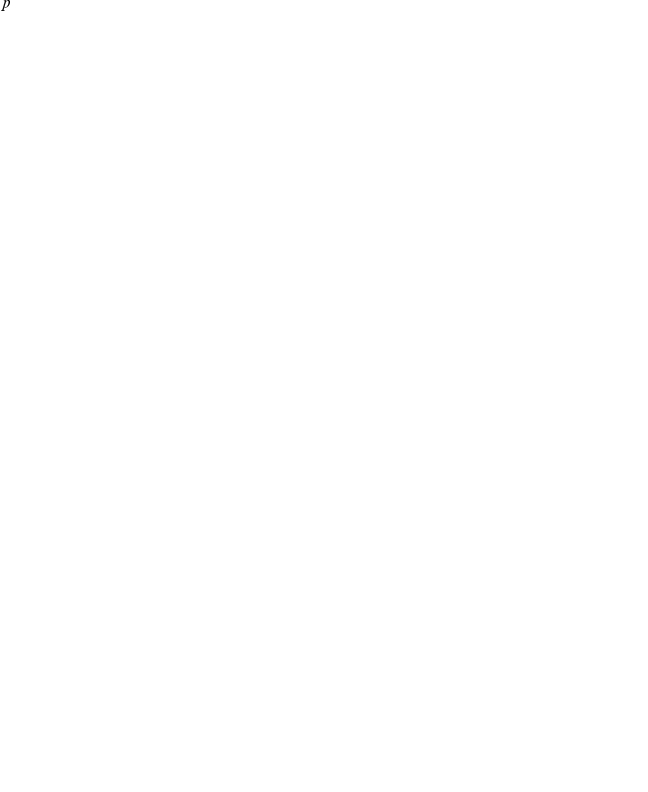
 gives the probability that the donor evaluates the recipient's trust score and with probability 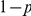 the donor evaluates the recipient's reputation score. In addition, a donor 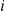 has her own assessment strategies 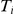 and 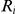, drawn from a uniform distribution in 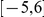, for trust and reputation respectively. Therefore, the model comprises 144 different strategies. According to whether the donor evaluates the recipient's trustworthiness or reputation, cooperation will be established if the recipient's image is above a certain threshold given by 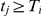 or 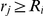 for trustworthiness and reputation respectively. If cooperation is established, the donor's reputation 
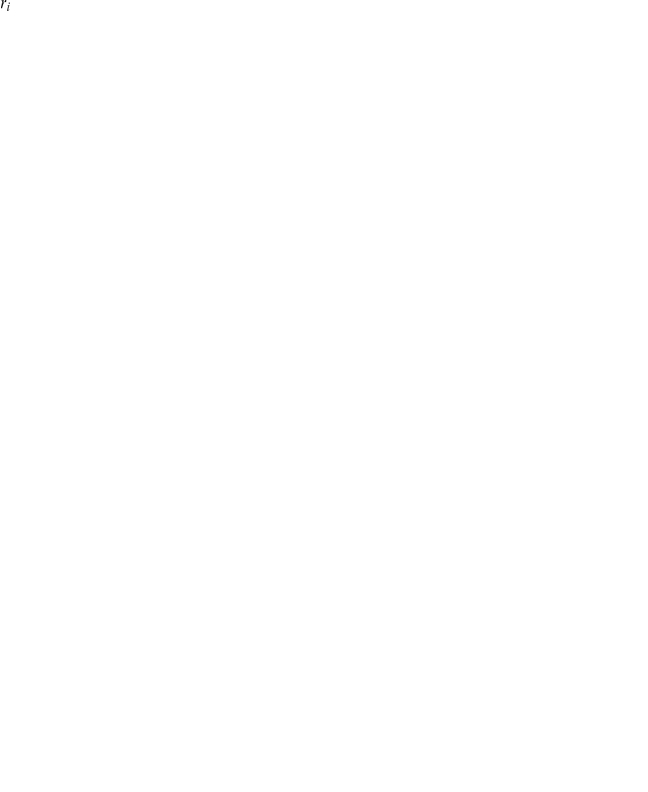
 is increased by one unit, else her reputation decreases by one unit. In addition, each time the recipient receives cooperation her trustworthiness 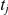 is increased by one unit, else her trustworthiness decreases by one unit. Note that the increase and decrease of scores is subject to the boundary conditions of the score values 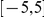. This score boundary allows the presence of unconditional cooperators 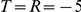 and unconditional defectors 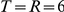. At the end of its lifetime, the population is replaced by a new generation, where an old actor 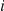 can transmit her assessment strategies T-R to a new actor 
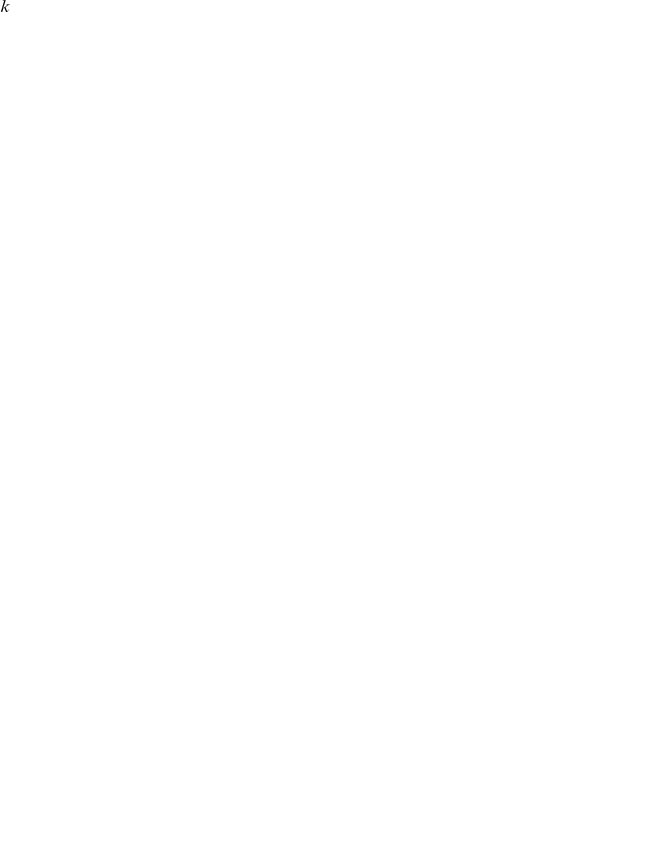
, with a probability 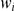 proportional to her own payoff and relative to the payoffs of all actors 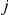 in the population [45]. Mathematically, this is given by 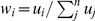, where 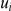 is the payoff of actor 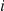. Since payoffs can be negative, we take the minimum value as the baseline payoff equal to 1 and adjust all other payoffs accordingly. If not stated otherwise, all generations start with 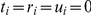 for all actors 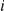. Simulations were performed using conventional parameter values [10], [11]: 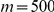, 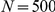, 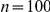, 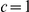 and 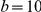. We also extended our model for large populations with up to 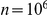 actors and found similar results.

### Share-alike mechanism

According to whether the donor evaluates the recipient's trust or reputation scores, cooperation will be established if the recipient's image is below a certain threshold given by 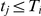 or 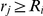 for trust and reputation respectively.
